# How to Improve the Predictions of Plant Functional Traits on Ecosystem Functioning?

**DOI:** 10.3389/fpls.2021.622260

**Published:** 2021-02-04

**Authors:** Congcong Liu, Ying Li, Pu Yan, Nianpeng He

**Affiliations:** ^1^Key Laboratory of Ecosystem Network Observation and Modeling, Institute of Geographic Sciences and Natural Resources Research, Chinese Academy of Sciences, Beijing, China; ^2^College of Resources and Environment, University of Chinese Academy of Sciences, Beijing, China; ^3^Key Laboratory of Vegetation Ecology, Ministry of Education, Institute of Grassland Science, Northeast Normal University, Changchun, China

**Keywords:** functional traits, functional diversity, intraspecific variability, trait selection, scale-matching, ecosystem traits

## Introduction

Functional traits are defined as morpho-physio-phenological traits that indirectly impact fitness via their effects on growth, reproduction, and survival (Violle et al., 2007). These traits can be further divided into response traits and effect traits. Response traits describe a plant's response to environmental change, while effect traits describe the effect of a plant on ecosystem functioning (Violle et al., 2007). According to the above definitions, a specific functional trait can reflect the adaptive strategy of plants or their impact on ecosystem function. Theoretically, plant functional traits can track environmental changes and play important roles in determining ecosystem functioning. Therefore, establishing the linkages between plant functional traits and ecosystem functioning has been one of the most commonly researched areas in ecology.

**Figure 1:**
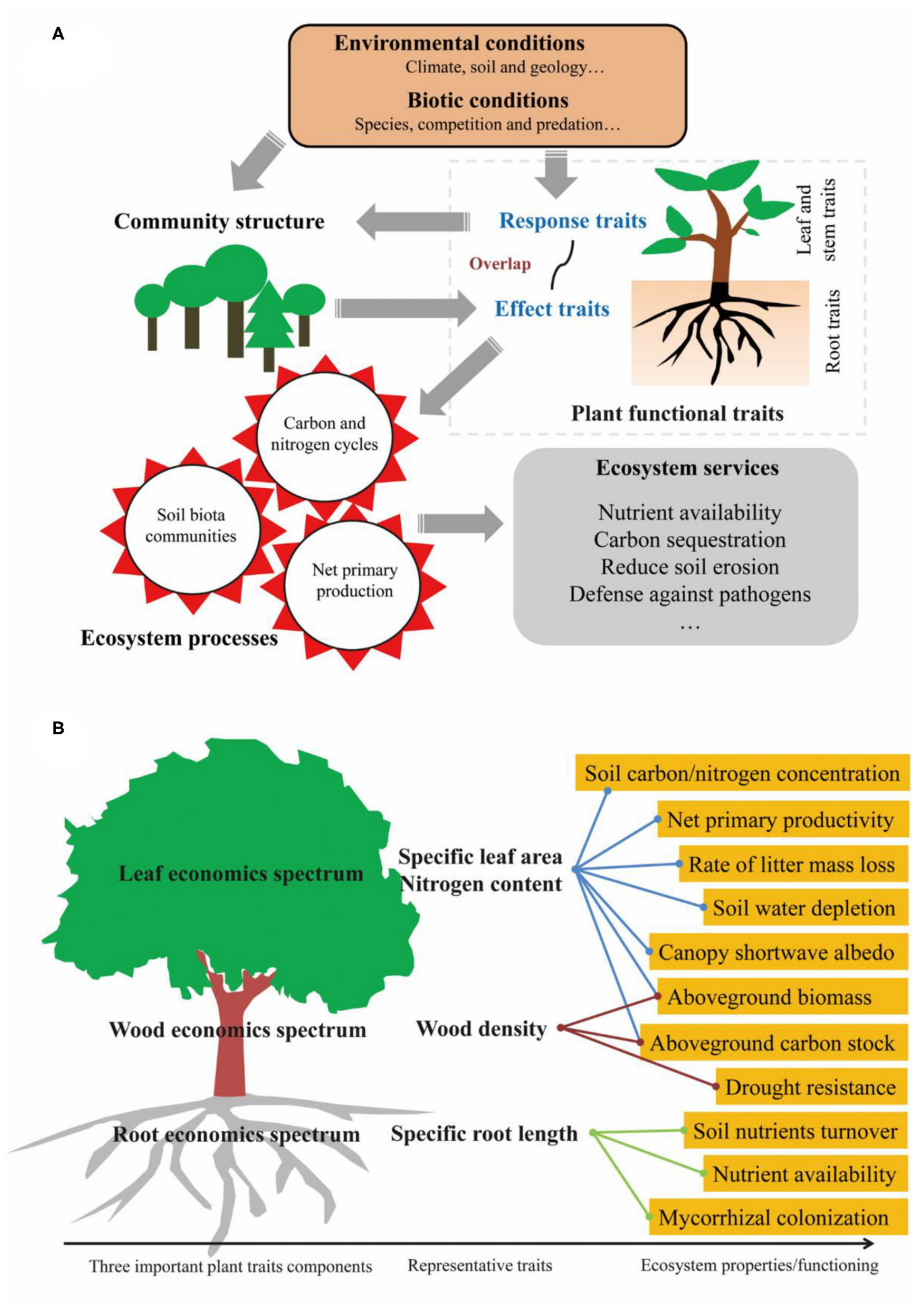
How plant functional traits determine ecosystem functioning **(A)** and some specific examples **(B)**. **(A)** is adapted from Lavorel and Garnier (2002) and Faucon et al. (2017).

There has been a growing consensus that plant functional traits strongly determine ecosystem functioning (DiAz and Cabido, 2001) and, based on previous studies (Lavorel and Garnier, 2002; Faucon et al., 2017), we present a framework to illustrate how plant functional traits determine ecosystem functioning (Figure 1A). Although many studies have reported strong linkages between functional traits and ecosystem functioning (Garnier et al., 2004), a recent study by van der Plas et al. (2020) states that plant functional traits are, in fact, poor predictors of ecosystem functioning. Thus, the relationships between functional traits and ecosystem functioning are controversial.

## Why Are There Controversies About Plant Functional Traits and Ecosystem Functioning?

In the study by van der Plas et al. (2020), 41 plant traits and 42 ecosystem properties/functioning in 78 experimentally manipulated grassland plots were measured over 10 years. This unprecedented dataset is used to test how plant traits predict ecosystem functioning. However, van der Plas et al. (2020) reported that plant traits are poor predictors of ecosystem properties. On the contrary, previous studies have established linkages between certain plant traits and ecosystem functions, such as specific leaf area and productivity (Violle et al., 2007), leaf nitrogen content and productivity (Reich, 2012), and chlorophyll content and productivity (Li et al., 2018). Why do all of these studies predict productivity spontaneously using leaf economic traits? As photosynthesis depends on leaf economic traits, strong correlations between leaf economic traits and ecosystem productivity are often observed.

Appropriate traits must be identified that explain a species' influence on ecosystem functioning (“effect traits”) and the response of those species to environmental change (“response traits”). A trait may correspond to one or several ecosystem functions (Lawren and Buckley, 2020), but it is not likely to correspond to them all. Here, we present a simple summary about the relationship between plant traits and ecosystem functions (Figure 1B), such as leaf economic traits (Wright et al., 2004), *e.g*., specific leaf area and leaf nitrogen content, are correlated with net primary productivity (Reich, 2012), soil carbon and nitrogen content (Lienin and Kleyer, 2012), rate of litter mass loss (Lienin and Kleyer, 2012), canopy shortwave albedo (Ollinger et al., 2008), and soil water depletion (Wilke and Snapp, 2008). Wood economic traits (Chave et al., 2009), *e.g*., wood density, are correlated with aboveground biomass, carbon stock (Bu et al., 2019), and drought resistance (Chao et al., 2008). Root economic traits (Mommer and Weemstra, 2012), *e.g*., specific root length, are correlated with soil nutrient turnover, nutrient availability, and mycorrhizal colonization (Guo et al., 2008; Bardgett et al., 2014; Freschet et al., 2020). All of these studies show that some specific above- and below-ground traits are involved in certain ecosystem processes and functioning; however, van der Plas et al. (2020) designed their study without giving this aspect due consideration.

Rational trait selection is vital for the prediction of ecosystem functioning, and arbitrarily linking plant traits to ecosystem properties confuses the real relationships between traits and ecosystem functioning. In other words, the traits should be selected according to their roles in the underlying mechanisms of those functions. Besides, the effects of plant traits on ecosystem functioning may not be observed within a short time; for example, the effects of plant traits on carbon sequestration in soils are only observable after 10 years or even longer (De Deyn et al., 2008), thus categorizing ecosystem properties according to temporal dynamics might be important. However, even doing this might be more challenging than expected.

## Discussion

Here, we list several achievable approaches to improve predictions of the effects of plant functional traits on ecosystem function under rational trait selection. This will help ecologists use plant functional traits as input parameters of dynamic global vegetation models to improve their precision (Yang et al., 2015).

### Individual-Level Trait Measurement

Intraspecific variability of functional traits might also have significant effects on ecosystem functioning (Albert et al., 2010). Generally, intraspecific variability of plant traits could be negligible compared with their interspecific variability; however, more and more evidence has shown that intraspecific functional variability can have significant effects on community dynamics and ecosystem functioning (Fu et al., 2020). For example, individual-level morphophysiological traits of phytoplankton can improve predictions on community resource-use and biomass yield (Fontana et al., 2018). Individual-level tree heights are strongly correlated with growth rate (Liu et al., 2016), and interspecific variation in specific root length drives aboveground biodiversity effects (Bu et al., 2017). It is, therefore, necessary to consider the intraspecific variability of functional traits to improve the predictive power of functional traits on ecosystem functioning.

### Functional Diversity Selection

#### Trait-Abundance Distributions for Singular Trait

Relationships between plant traits and ecosystem properties may not be as strong as expected if only traditional community-weighted methods are used. Trait-abundance distributions may offer a promising solution to the above problem. Trait-abundance distributions describe the shape of the frequency distribution of plant traits in a specific community and include the community-weighted mean, variance, skewness, and kurtosis. The variance, skewness, and kurtosis compensate for a weakness associated with the mean in terms of its potential to mistakenly exacerbate the role of dominant species (Liu et al., 2020). The variance is the functional divergence, skewness signifies the extent of asymmetric distribution of traits, and kurtosis signifies functional evenness. Trait-abundance distributions of phenological traits could help us quantify the asynchrony of plant growth and development within communities. Thus, trait-abundance might improve the predictive power of plant traits for ecosystem properties.

Skewness and kurtosis for specific leaf area and maximum plant height have been found to explain 38% of the variation in multifunctionality (Gross et al., 2017). This is greater than the variance (15%) explained by the mean and variance of these two traits. Such results show that models that include the skewness-kurtosis of trait-abundance distributions have a much higher explanatory power in terms of multifunctionality than models that include only the mean and variance of the trait-abundance distribution.

#### Functional Diversity Indices for Multiple Traits

Functional diversity is increasingly identified as an important driver of ecosystem functioning (Villéger et al., 2008), and Song et al. (2014) reviewed the relationships between functional diversity and ecosystem functioning. It is generally accepted that functional diversity consists of three independent components—functional richness, functional evenness, and functional divergence. However, a wide variety of algorithms are generated to delineate the trait space occupied by species, each based on particular mathematical objects, such as distance, hypervolume, and others (Laliberté and Legendre, 2010; Swanson et al., 2015; Junker et al., 2016). Under rational trait selection, functional diversity indices and their algorithms require careful deliberation to improve their predictions for ecosystem functioning.

### Scale-Matching Between Traits and Ecosystem Properties

In most cases, functional identity and diversity are, in terms of scale, mismatched with ecosystem properties. This situation occurs because most ecosystem properties are based on unit ground area, whereas most plant traits (or leaf traits) are based on unit leaf area or mass. This mismatch may hinder the process of linking plant traits with ecosystem properties. Ecosystem traits are traits expressed in terms of intensity (or density) normalized per unit ground area. This mode of expression is not bound by the limits imposed by the units of measurement used for plant traits and ecosystem properties. Using ecosystem traits could address the challenges associated with broadening the predictive power of plant traits in relation to ecosystem properties (He et al., 2019). For example, stomata are pores on leaves through which water vapor and carbon dioxide diffuse, so stomatal traits are “appropriate traits” for ecosystem productivity. By scaling individual stomatal traits up to the community level based on ground area, stomatal density can explain 51% of the total variation in forest ecosystem productivity on a large scale (Wang et al., 2015). This finding indicates that scale-matching is an important factor in improving ecosystem property predictions using plant traits.

## Conclusion

In summary, not all traits are suitable for use in predicting specific ecosystem properties. The procedure “Rational-trait selection–Individual-level trait measurement–Functional diversity selection–Scale-matching between traits and ecosystem properties” may improve the predictive power of plant functional traits on ecosystem functioning. Besides, the fact that plant traits are widely measured does not mean that they are of key functional importance. Phenological traits, the plant development stage, and the asynchrony of plant growth for coexisting species, and “hard” traits, which are more closely related to a precise function but difficult to measure, should also attract attention in the future.

## Author Contributions

NH conceived the ideas. CL, PY, and YL led the writing of the manuscript. All authors contributed critically to the drafts and gave final approval for publication.

## Conflict of Interest

The authors declare that the research was conducted in the absence of any commercial or financial relationships that could be construed as a potential conflict of interest.

## References

[B1] Albert C. H. Thuiller W. Yoccoz N. G. Douzet R. Aubert S. Lavorel S. (2010). A multi-trait approach reveals the structure and the relative importance of intra- vs. interspecific variability in plant traits. Funct. Ecol. 24, 1192–1201. 10.1111/j.1365-2435.2010.01727.x

[B2] Bardgett R. D. Mommer L. De Vries F. T. (2014). Going underground: root traits as drivers of ecosystem processes. Trends Ecol. Evol. 29, 692–699. 10.1016/j.tree.2014.10.006 25459399

[B3] Bu W. Schmid B. Liu X. Li Y. Härdtle W. von Oheimb G. (2017). Interspecific and intraspecific variation in specific root length drives aboveground biodiversity effects in young experimental forest stands. J. Plant Ecol. 10, 158–169. 10.1093/jpe/rtw096

[B4] Bu W. S. Zhang C. C. Huang J. H. Zang R. G. Ding Y. Xu H. (2019). The influences of disturbance histories and soil properties on aboveground biomass through plant functional traits in a tropical rainforest. Forests 10, 1–7. 10.3390/F10090774

[B5] Chao K.-J. Phillips O. L. Gloor E. Monteagudo A. Torres-Lezama A. Martínez R. V. (2008). Growth and wood density predict tree mortality in Amazon forests. J. Ecol. 96, 281–292. 10.1111/j.1365-2745.2007.01343.x

[B6] Chave J. Coomes D. Jansen S. Lewis S. L. Swenson N. G. Zanne A. E. (2009). Towards a worldwide wood economics spectrum. Ecol. Lett. 12, 351–366. 10.1111/j.1461-0248.2009.01285.x 19243406

[B7] De Deyn G. B. Cornelissen J. H. Bardgett R. D. (2008). Plant functional traits and soil carbon sequestration in contrasting biomes. Ecol. Lett. 11, 516–531. 10.1111/j.1461-0248.2008.01164.x 18279352

[B8] DiAz S. Cabido M. (2001). Vive la différence: plant functional diversity matters to ecosystem processes. Trends Ecol. Evol. 16, 646–655. 10.1016/S0169-5347(01)02283-2

[B9] Faucon M.-P. Houben D. Lambers H. (2017). Plant functional traits: Soil and ecosystem services. Trends Plant Sci. 22, 385–394. 10.1016/j.tplants.2017.01.005 28209328

[B10] Fontana S. Thomas M. K. Moldoveanu M. Spaak P. Pomati F. (2018). Individual-level trait diversity predicts phytoplankton community properties better than species richness or evenness. ISME J. 12, 356–366. 10.1038/ismej.2017.160 28972571 PMC5776449

[B11] Freschet G. T. Roumet C. Comas L. H. Weemstra M. Bengough A. G. Rewald B.. (2020). Root traits as drivers of plant and ecosystem functioning: current understanding, pitfalls and future research needs. New Phytol. 10.1111/nph.17072. [Epub ahead of print]. 33159479

[B12] Fu H. Yuan G. Jeppesen E. (2020). Trait-based community assembly of submersed macrophytes subjected to nutrient enrichment in freshwater lakes: Do traits at the individual level matter? Ecol. Indic. 110:105895 10.1016/j.ecolind.2019.105895

[B13] Garnier E. Cortez J. Billès G. Navas M.-L. Roumet C. Debussche M. (2004). Plant functional markers capture ecosystem properties during secondary succession. Ecology 85, 2630–2637. 10.1890/03-0799

[B14] Gross N. Bagousse-Pinguet Y. L. Liancourt P. Berdugo M. Gotelli N. J. Maestre F. T. (2017). Functional trait diversity maximizes ecosystem multifunctionality. Nat. Ecol. Evolut. 1:0132. 10.1038/s41559-017-0132 28497123 PMC5421574

[B15] Guo D. Xia M. Wei X. Chang W. Liu Y. Wang Z. (2008). Anatomical traits associated with absorption and mycorrhizal colonization are linked to root branch order in twenty-three Chinese temperate tree species. New Phytol. 180, 673–683. 10.1111/j.1469-8137.2008.02573.x 18657210

[B16] He N. Liu C. Piao S. Sack L. Xu L. Luo Y. (2019). Ecosystem traits linking functional traits to macroecology. Trends Ecol. Evol. 34, 200–210. 10.1016/j.tree.2018.11.004 30527959

[B17] Junker R. R. Kuppler J. Bathke A. C. Schreyer M. L. Trutschnig W. (2016). Dynamic range boxes – a robust nonparametric approach to quantify size and overlap of n-dimensional hypervolumes. Methods Ecol. Evolut. 7, 1503–1513. 10.1111/2041-210X.12611

[B18] Laliberté E. Legendre P. (2010). A distance-based framework for measuring functional diversity from multiple traits. Ecology 91, 299–305. 10.1890/08-2244.1 20380219

[B19] Lavorel S. Garnier E. (2002). Predicting changes in community composition and ecosystem functioning from plant traits: revisiting the Holy Grail. Funct. Ecol. 16, 545–556. 10.1046/j.1365-2435.2002.00664.x

[B20] Lawren S. Buckley T. N. (2020). Trait multi-functionality in plant stress response. Integr. Comp. Biol. 60, 98–112. 10.1093/icb/icz152 31825509

[B21] Li Y. Liu C. Zhang J. Yang H. Xu L. Wang Q. (2018). Variation in leaf chlorophyll concentration from tropical to cold-temperate forests: association with gross primary productivity. Ecol. Indic. 85, 383–389. 10.1016/j.ecolind.2017.10.025

[B22] Lienin P. Kleyer M. (2012). Plant trait responses to the environment and effects on ecosystem properties. Basic Appl. Ecol. 13, 301–311. 10.1016/j.baae.2012.05.002

[B23] Liu C. Li Y. Zhang J. Baird A. S. He N. (2020). Optimal community assembly related to leaf economic- hydraulic-anatomical traits. Front. Plant Sci. 11:341. 10.3389/fpls.2020.00341 32269584 PMC7109333

[B24] Liu X. Swenson N. G. Lin D. Mi X. Umaña M. N. Schmid B.. (2016). Linking individual-level functional traits to tree growth in a subtropical forest. Ecology 97, 2396–2405. 10.1002/ecy.1445 27859093

[B25] Mommer L. Weemstra M. (2012). The role of roots in the resource economics spectrum. New Phytol. 195, 725–727. 10.1111/j.1469-8137.2012.04247.x 22861183

[B26] Ollinger S. V. Richardson A. D. Martin M. E. Hollinger D. Y. Frolking S. E. Reich P. B.. (2008). Canopy nitrogen, carbon assimilation, and albedo in temperate and boreal forests: Functional relations and potential climate feedbacks. Proc. Natl. Acad. Sci. U.S.A. 105, 19336–19341. 10.1073/pnas.0810021105 19052233 PMC2593617

[B27] Reich P. B. (2012). Key canopy traits drive forest productivity. Proc. R. Soc. B: Biol. Sci. 279, 2128–2134. 10.1098/rspb.2011.2270 22279168 PMC3321697

[B28] Song Y. Wang P. Li G. Zhou D. (2014). Relationships between functional diversity and ecosystem functioning: a review. Acta Ecol. Sinica 34, 85–91. 10.1016/j.chnaes.2014.01.001

[B29] Swanson H. K. Lysy M. Power M. Stasko A. D. Johnson J. D. Reist J. D. (2015). A new probabilistic method for quantifying n-dimensional ecological niches and niche overlap. Ecology 96, 318–324. 10.1890/14-0235.1 26240852

[B30] van der Plas F. Schröder-Georgi T. Weigelt A. Barry K. Meyer S. Alzate A.. (2020). Plant traits alone are poor predictors of ecosystem properties and long-term ecosystem functioning. Nat. Ecol. Evol. 4, 1602–1611. 10.1038/s41559-020-01316-9 33020598

[B31] Villéger S. Mason N. W. Mouillot D. (2008). New multidimensional functional diversity indices for a multifaceted framework in functional ecology. Ecology 89, 2290–2301. 10.1890/07-1206.1 18724739

[B32] Violle C. Navas M.-L. Vile D. Kazakou E. Fortunel C. Hummel I. (2007). Let the concept of trait be functional! Oikos 116, 882–892. 10.1111/j.0030-1299.2007.15559.x

[B33] Wang R. Yu G. He N. Wang Q. Zhao N. Xu Z.. (2015). Latitudinal variation of leaf stomatal traits from species to community level in forests: linkage with ecosystem productivity. Sci. Rep. 5:14454. 10.1038/srep14454 26403303 PMC4585881

[B34] Wilke B. J. Snapp S. S. (2008). Winter cover crops for local ecosystems: linking plant traits and ecosystem function. J. Sci. Food Agric. 88, 551–557. 10.1002/jsfa.3149

[B35] Wright I. J. Reich P. B. Westoby M. Ackerly D. D. Baruch Z. Bongers F. (2004). The worldwide leaf economics spectrum. Nature 428, 821–827. 10.1038/nature02403 15103368

[B36] Yang Y. Zhu Q. Peng C. Wang H. Chen H. (2015). From plant functional types to plant functional traits: a new paradigm in modelling global vegetation dynamics. Prog. Phys. Geograph. Earth Environ. 39, 514–535. 10.1177/0309133315582018

